# An increase in livestock density increases forage nutritional value but decreases net primary production and annual forage nutritional yield in the alpine grassland of the Qinghai-Tibetan Plateau

**DOI:** 10.3389/fpls.2022.1020033

**Published:** 2022-11-24

**Authors:** Xixi Yao, Changhui Li, Anum Ali Ahmad, Akash Tariq, A. Allan Degen, Yanfu Bai

**Affiliations:** ^1^ State Key Laboratory of Plateau Ecology and Agriculture, College of Agriculture and Animal Husbandry, Qinghai University, Xining, China; ^2^ Lanzhou Institute of Husbandry and Pharmaceutical Sciences, Chinese Academy of Agricultural Sciences, Lanzhou, China; ^3^ Xinjiang Key Laboratory of Desert Plant Roots Ecology and Vegetation Restoration, Xinjiang Institute of Ecology and Geography, Chinese Academy of Sciences, Urumqi, China; ^4^ Desert Animal Adaptations and Husbandry, Wyler Department of Dryland Agriculture, Blaustein Institutes for Desert Research, Ben-Gurion University of the Negev, Beer Sheva, Israel; ^5^ College of Grassland Science and Technology, Sichuan Agriculture University, Chengdu, China

**Keywords:** alpine grasslands, herbivore densities, climate change, dominant plant species, pasture

## Abstract

Pasture biomass and quality are dependent on herbivore grazing and precipitation, but the responses of vegetation to the interactive effects of climate and grazing regimes remain unclear. We conducted an eight-year sheep grazing experiment with 4 stocking rates (0, 3.5, 5.5, and 7.5 sheep/ha) in an alpine meadow of the northeastern Tibetan Plateau. The above-ground net primary productivity (ANPP) and forage nutritional value (FNV) of four dominant species *(Poa annua, Kobresia humilis, Astragalus adsurgens* and *Potentilla fruticosa)* were measured during a wet year (360 mm rainfall) and a drought year (216 mm rainfall). The FNV was used as indicator of forage quality and was calculated from the crude protein (CP) content, *in vitro* true dry matter digestibility (IVTD), metabolic energy (ME) yield, and neutral detergent fiber (NDF) content of the plant. The stocking rate explained a minimum of 76% of the variations of ANPP, and the precipitation sub-additive effect for ANPP ranged from 5% to 12%. The interaction of sheep stocking rate and precipitation affected ANPP of the 4 species, except for *P. fruticosa.* The FNV of the pasture increased with increasing grazing pressure, but ANPP and forage nutritional yield (FNY) decreased. In calculating FNY, the increase in FNV did not compensate for the decrease in ANPP. In non-grazed plots, the CP yield declined sharply (18%-55%) in response to drought, but there was no effect on ME yield. The interaction between stocking rate and precipitation affected forage quality of the 4 plant species differently. The grassland ANPP and FNY could be maintained at a grazing intensity of 3.5 sheep/ha in wet and dry years. Our results highlight that stocking density affects pasture ANPP and FNV, and is contingent on rainfall.

## Introduction

The Qinghai-Tibetan Plateau (QTP), regarded as the world’s roof, is the highest (elevation 4000 m above sea level on average) and largest (2.57 million km^2^, 25% of China’s total area) plateau ecosystem (Cao et al., 2011; Lin et al., 2011). Approximately 85% of the QTP is alpine meadow, which provides important ecological services and supports China’s grassland animal production (Pei et al., 2000; Mysterud et al., 2001; He et al., 2009; Wan et al., 2011; Jing et al., 2013). However, overgrazing has contributed to the degradation and loss of pasture biomass of the QTP (Harris, 2010; Cheng et al., 2016). Approximately 90% of alpine grassland has been degraded, with 35% seriously degraded into a “black-soil-type grassland” (Jing et al., 2013; Dong & Sherman, 2015). The degradation of alpine grasslands has become a crucial issue in China (Xu & Zhou, 2005; Jing et al., 2013), and, consequently, ecosystem management and restoration of alpine grassland have emerged as immediate needs in government policies (Bai et al., 2004; Jiang et al., 2012; Cheng et al., 2016).

Traditionally, livestock such as yaks and Tibetan sheep graze all year round, with no feed supplements provided. Grazing can affect the annual forage nutritive yield (FNY), which is determined by above-ground net primary productivity (ANPP) and forage nutritive value (FNV), and is the main constraint to livestock production (Du et al., 2004; Ren et al., 2008). To assess the effects of grazing on FNV, recent research has focused on the direct effects of biomass removal and the indirect effects of changes in soil nutrient availability. Grazing or pasture biomass removal promotes the regeneration of pasture and improves the nutritive value and digestibility of the pasture (Ren et al., 2016). The plant regeneration potential depends on phenology and soil nutrients and water content, which are affected by precipitation (Van Soest et al., 1991; Schulze et al., 1994; Gebauer & Ehleringer, 2000; Miao et al., 2015). In addition: (1) livestock grazing reduces vegetation coverage and alters the soil water content and soil temperature, which affect the mineralization of nitrogen and the availability of soil nutrients (Snyman, 2002); and, (2) faeces and urine promote nitrogen and phosphorus cycling and increases the availability of soil nutrients (Giese et al., 2011). These changes in soil nutrient availability are affected by precipitation (Snyman, 2002; Giese et al., 2011).

In the present 8-year field study, we examined the effects of grazing density on the ANPP and FNV of 4 dominant plant species in an alpine grassland of the QTP. By employing four sheep grazing intensities (stocking rates), the following questions were addressed: (1) how does ANPP and FNV of dominant plant species in the alpine grassland of the QTP respond to stocking rate? and 2) how is ANPP and FNV affected by a wet or dry year?

## Materials and methods

### Study site

**Figure 1:**
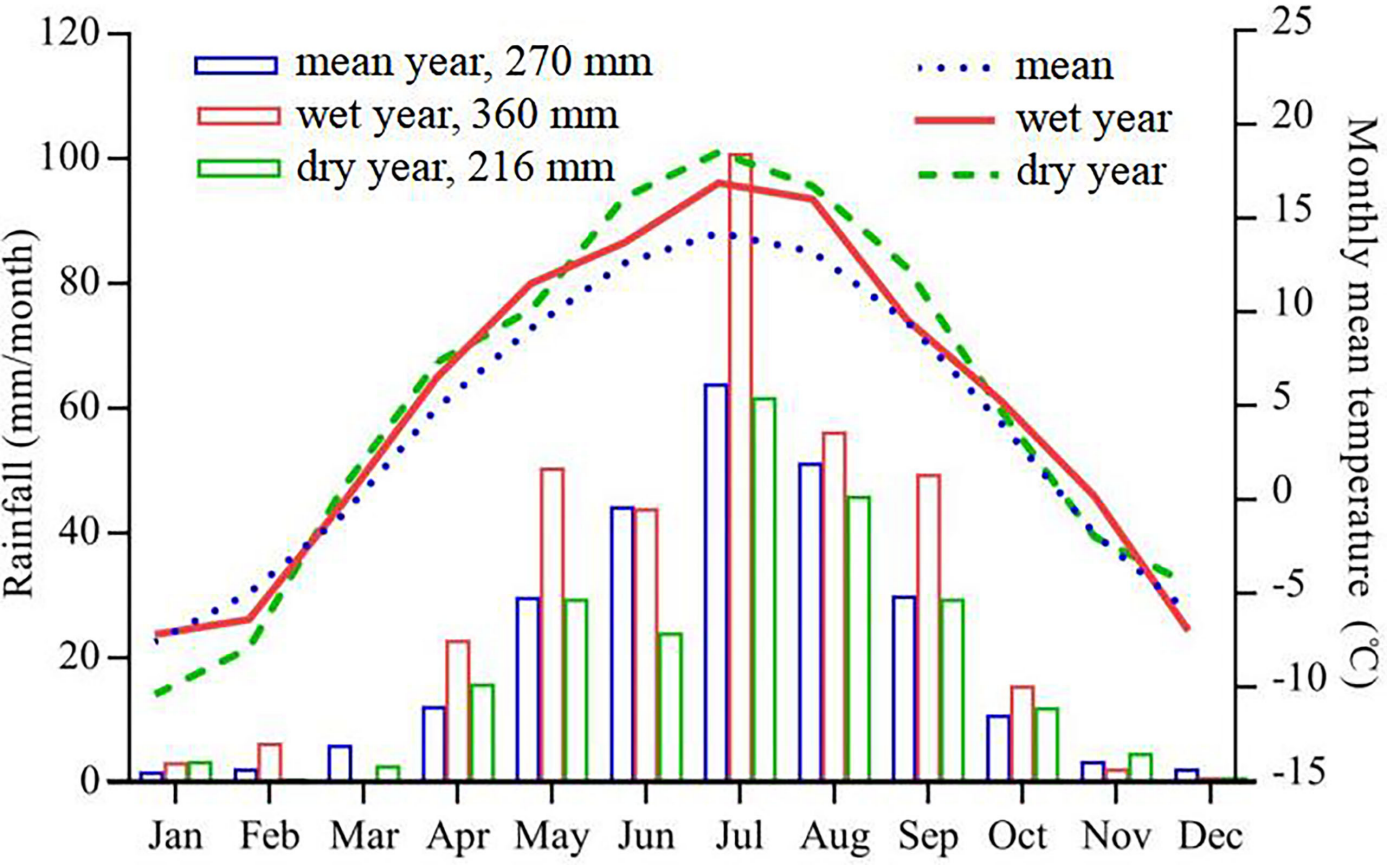
The monthly temperature (right Y-axis), and rainfall ( left Y-axis) at the study site on the alpine grassland over 34 years (1985-2018).

This study was carried out on the northeastern edge of the Tibetan Plateau, Gannan Tibetan Autonomous Prefecture of Gansu Province, in the PR of China (102°18′56”E, 34°28′53”N; 3540 m above sea level). Annual precipitation averaged 270 mm, and air temperature averaged 1.7°C over 34-years (1985-2018) (**Figure 1** right Y-axis). The precipitation in 2019 was 360 mm (wet year, 33% higher than the 34-year average) and in 2020 was 216 mm (dry year, 20% lower than the 34-year average), with ~85% occurring during the growing season (May-September) (**Figure 1** left Y-axis). The type of grassland is alpine and the soil is black meadow. The plant growth period is short, about 120 days from June to September, and the dominant species include a perennial Gramineae (*Poa annua*), sedge (*Kobresia humilis*), legume (*Astragalus adsurgens*), and Rosaceae (*Potentilla fruticosa*).

### Experimental design

The entire study area was degraded slightly and was traditionally used as summer pasture, being grazed from June to September by Tibetan sheep. Four stocking rates were established, namely, 0, 3.5, 5.5, and 7.5 sheep/ha, each in an enclosed 4-ha area. There were three replicates for each stocking rate for a total of 12 enclosures, which were distributed randomly over a homogeneous area of 48 ha, and with at least 100 m separating any two enclosures. All enclosures were similar with a 9° slope on the sunny side. There were three 100 m × 100 m monitoring plots in each replicate, with at least a 50 m separation between plots, for a total of 36 plots (4 stocking rates × 3 replicates × 3 plots). All sites were fenced year-round since 2010 and were grazed at the designed stocking rate from June 10 to September 10. The plots for the stocking rate of 0 sheep/ha (non-grazed control) were fenced and not grazed. Female Tibetan sheep, aged 20 months and approximately 35 kg live weight, were chosen randomly at the start of each year from sheep herded by local farmers. Management of the Tibetan sheep followed traditional practice, with sheep kept at the grazing sites both day and night, and drinking water freely available. The site was owned by a local farmer who agreed to its use for this study. There was no endangered or protected species within the study area.

### Pasture sampling

Pasture samples were collected on the 15th day of each month from June to September in 2019 (the wet year) and 2020 (the dry year). Five random quadrats (1 m ×1 m) were selected in every plot, with at least 1 m between any two quadrats to eliminate marginal effects. The number of species and the family, coverage and above-ground biomass for each species were recorded in each quadrat. Each plant was clipped to 1-cm from the ground, placed into a separate paper bag according to species, and transported to the laboratory. The plants were dried at 105°C for 30 min, and then maintained at 60°C for 48 h to determine above-ground biomass of each plant species.

### Chemical analyses

To assess the nutritive value, the four oven-dried plant species were cut into 1-2 cm pieces using scissors, and then ground (stereo metric formula feed mills BO-1000S2) to pass through a 1-mm sieve. Forage nutritional value (FNV) was based on the concentrations of crude protein (CP) and neutral detergent fibre (NDF), the *in vitro* true dry matter digestibility (IVTD) and metabolizable energy (ME) yield of each of the 4 dominant species. Nitrogen (N) concentration was measured using a fully automated Kjeldahl analyzer (Kjeltec 8400, Foss, Hilleroed, Denmark) (Feldsine et al., 2002) and NDF was measured using an automated fiber analyzer (ANKOM 2000, Macedon, NY, USA) (Van Soest, 1994; Schönbach et al., 2009).

The IVTD of forage was measured using an artificial rumen simulation incubator (Daisy*^PIIP^* Incubator, ANKOM, Macedon, NY, USA). At 07:00, rumen fluid was collected using an oral-stomach tube from five fasting female Tibetan sheep aged 20 months. Approximately 300 mL of rumen fluid were collected from each sheep, of which the first 50 mL were discarded to avoid contamination from saliva. Total fluid collection was completed in 30 min. The rumen fluid was placed immediately into a preheated thermos at 39°C, filled with CO_2_, and stoppered. The rumen fluid was mixed and filtered through 4 layers of gauze. Rumen nutrient solution was prepared (Menke and Steingass, 1988), which was mixed with the rumen fluid at a volume ratio of 2:1, forming the culture solution. Three replicates were measured for each pasture sample. One g (to 0.001 g) of each replicate was weighed (W_i_) into an Ankom F57 filter bag, and, along with 3 empty filter bags as blanks, were placed into a fermenter. Four fermenters were cultured in the same batch, and then the fermenters were placed in an *in vitro* incubator preheated to 39°C. Subsequently, CO_2_ was pumped into the incubator to replace the oxygen, and 1600 mL of preheated culture medium at 39°C were added. After 48 h of fermentation, the filter bags were removed and quickly put into cold water to stop fermentation. The filter bags were rinsed gently with 39°C warm water and then placed on a disk to be dried until constant weight (W_f_).

The IVTD of the plant sample was calculated as: IVTD (%) = 100 × (W_i_ - W_f_)/W_i_ (Tilley & Terry, 1963).

Metabolizable energy (ME) yield in forage was estimated using the equation recommended by CSIRO (2007): ME = 0.17 × IVTD – 2, where ME is metabolizable energy (MJ/kg DM) and IVTD is *in vitro* true dry matter digestibility (%).

Forage nutritional value (FNV) index was based on CP content (g/100 g DM), ME (metabolizable energy (MJ/kg DM), IVTD (g/100 g DM), and NDF content (g/100 g DM) and calculated as:


FNV=(CP+ME+0.25 IVTD)–0.25 NDF

Forage nutritional yield (FNY) is a function of ANPP and FNV and was calculated as,


FNY=ANPP×FNV

### Statistical analyses

We used the Mixed Model in SPSS version 19.0 (IBM Corp., Armonk, NY, USA), based on an autoregressive covariance structure through ANOVA, to analyze the data. There were 1440 observations (4 stocking rates × 3 replicates × 3 plots × 5 quadrats × 2 years × 4 months) for each herbage variable (FNV, CP, IVTD, ME, and NDF). Repeated measure analyses for forage nutritive values used a mixed model, including stocking rates, precipitation (wet, dry) and plot as fixed effects with month (June, July, August and September) as a repeated effect, and their interactions. *P <* 0.05 was accepted as the level for significance and the Tukey’s test was used to separate means where significance was found.

## Results

### Effect of stocking rate on above-ground net primary productivity of forage

**Figure 2:**
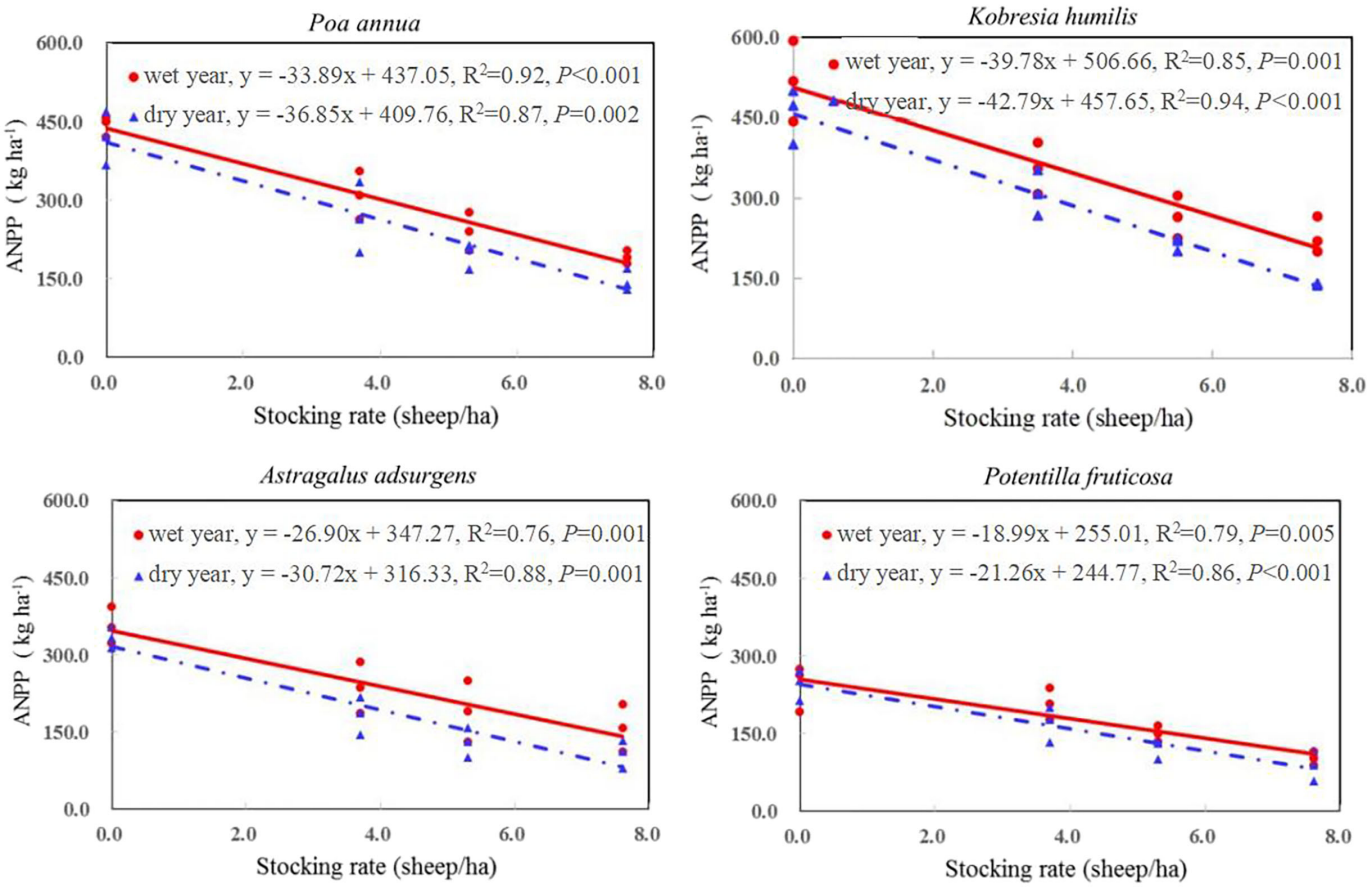
Response of above-ground net primary productivity (ANPP) of four dominant plant species to stocking rate in a wet year (2019) and a dry year (2020). Adjusted R^2^ and significance levels of the linear regressions are presented.

The species composition, plant families, and ANPP (kg/ha) were similar in mid-August of the wet (2019) year and dry year (2020) (**Table 1**), when ANPP peaked. The four dominant species contributed 88.6% - 89.3% of the ANPP in the wet year, and 88.5% - 90.2% of the ANPP in the dry year, with no difference among the four stocking rates (**Table 1**). The slope of the regression line of ANPP on stocking rate was greatest for *K. humilis*, with the slopes greater in the dry year than the wet year for all four species (**Figure 2**).

**Table 1:** Species and average above-ground biomass (kg/ha) for each plant species at each stocking rate in a wet year (2019) and a dry year (2020).

Species	Family	Above-ground biomass (kg/ha)							
		Wet year				Dry year			
		Stocking rate (sheep/ha)				Stocking rate (sheep/ha)			
		0	3.5	5.5	7.5	0	3.5	5.5	7.5
*Poa annua*	*Gramineae*	442.9	309.6	241.7	191.4	419.2	266.0	195.9	146.2
*Kobresia humilis*	*Cyperaceae*	518.3	355.4	264.4	228.2	457.5	308.9	215.1	138.8
*Scirpus triqueter*	*Cyperaceae*	15.7	12.1	7.6	2.7	8.8	4.3	6.6	0.9
*Carex atrata*	*Cyperaceae*	10.4	8.4	4.0	2.5	0.0	4.0	6.2	2.4
*Astragalus adsurgens*	*Leguminosae*	357.0	236.7	191.1	157.6	333.4	183.2	130.3	108.6
*Medicago ruthenica*	*Leguminosae*	17.9	6.9	0.5	3.2	13.9	6.2	9.9	2.5
*Gueldenstaedtia multiflora*	*Leguminosae*	15.2	11.3	8.8	4.3	16.9	10.6	8.6	2.3
*Melissitus ruthenicus*	*Leguminosae*	12.4	8.1	7.6	2.4	18.3	7.5	3.4	4.0
*Oxytropis kansuensis*	*Leguminosae*	12.0	11.4	8.4	6.4	9.2	5.8	5.2	5.3
*Potentilla fruticosa*	*Rosaceae*	244.3	208.2	150.0	102.2	245.6	170.0	122.7	87.8
*Potentilla bifurca*	*Rosaceae*	16.8	11.4	16.8	7.8	16.8	11.8	10.8	6.8
*Rheum pumilum*	*Polygonaceae*	16.4	10.7	5.2	4.4	12.4	8.0	1.2	1.8
*Polygonum macrophyllum*	*Polygonaceae*	8.8	7.2	8.4	5.5	8.4	8.4	8.4	4.6
*Leontopodium alpinum*	*Asteraceae*	15.3	9.7	6.7	3.3	11.3	7.0	0.0	2.0
*Taraxacum mongolicum*	*Asteraceae*	5.2	7.4	6.8	3.5	6.4	4.5	4.8	6.0
*Artemisia hedinii*	*Asteraceae*	10.8	9.6	4.4	14.4	6.8	7.2	4.8	2.4
*Gentianella pygmaea*	*Gentianaceae*	12.7	10.0	8.4	4.6	10.7	6.6	0.0	3.5
*Gentiana macrophylla*	*Gentianaceae*	4.8	4.4	4.0	9.2	4.8	6.0	2.4	4.0
*Geranium wilfordii*	*Geraniaceae*	2.8	2.4	3.2	6.4	8.8	4.4	2.0	2.9
*Pedicularis ikomai Sasaki*	*Scrophulariaceae*	4.4	3.6	2.4	10.4	3.2	0.0	1.6	3.4
*Thalictrum aquilegifolium*	*Ranunculaceae*	13.6	8.6	8.4	3.6	8.4	4.4	8.4	2.2
*Salix oritrepha*	*Salicaceae*	19.6	14.8	8.4	6.0	8.8	6.0	6.6	2.8
Total biomass (kg/ha)		1777.2	1267.8	967.3	780.1	1629.5	1040.8	754.9	541.2
The proportion of four dominant species (%)		88.6	89.0	89.3	88.7	89.2	89.2	88.5	90.2

### Inter-annual patterns of grazing effects on forage nutritional value

**Figure 3:**
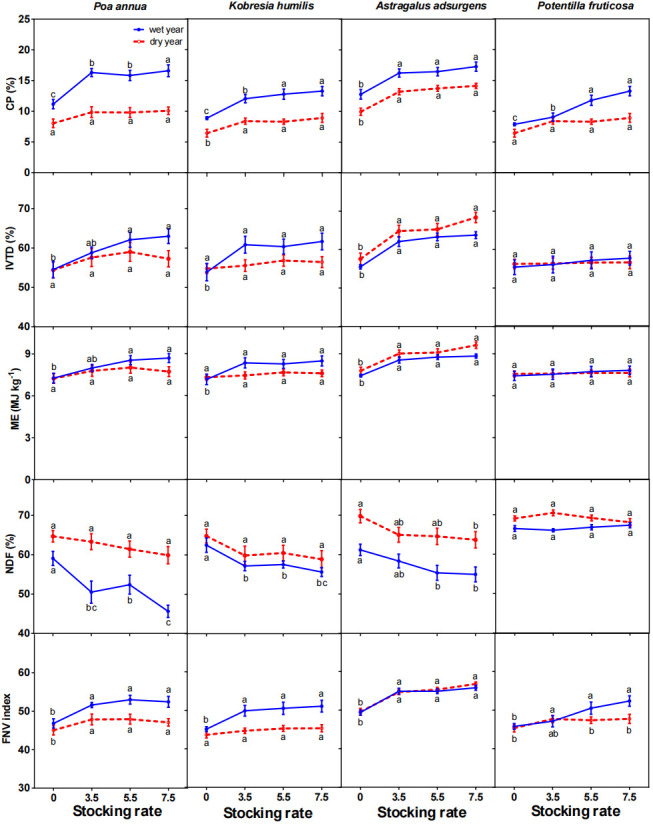
Effects of stocking rate on forage crude protein (CP) content, *in vitro* true digestibility (IVTD), metabolizable energy (ME), neutral detergent fiber (NDF) content and forage nutritional value (FNV) of four dominant species in a wet year (2019) and a dry year (2020) (mean ± standard error). Bars with different letters in the same year differ from each other (*P<* 0.05).

The FNV index in the wet year averaged 50.7 and in the dry year averaged 48.2 and increased in both years as stocking rate increased. In general, CP, IVTD, ME and FNV of the four dominant plant species increased, whereas NDF decreased as stocking rate increased (**Figure 3**; **Table S1** , **S2**), with the largest increase or decrease in the 7.5 sheep/ha in both the wet and dry years. The CP in the wet year was substantially higher than in the dry year for all four species. The slopes of the regression lines for CP and NDF on stocking rates were greatest for *K. humilis* in the wet year and for *P. annua* in the dry year (**Table S2**); whereas, the slopes for the regression lines of IVTD and ME on stocking rate were greatest for *P. annua* in the wet year ( **Table S2**).

### Seasonal patterns of grazing effects on forage nutritional value

**Figure 4:**
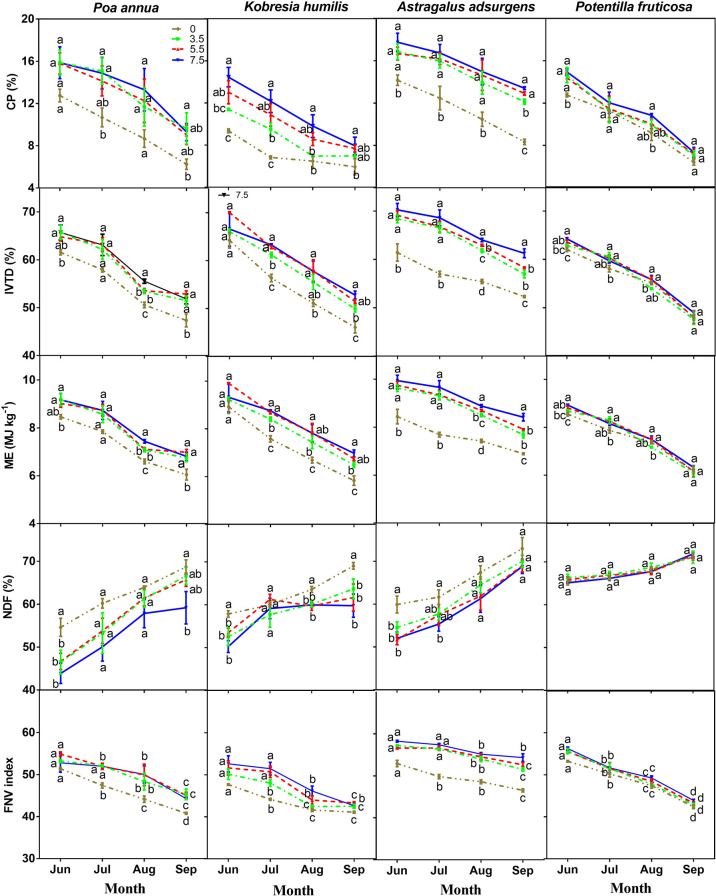
Effects of stocking rate on crude protein (CP) content, *in vitro* true digestibility (IVTD), metabolizable energy (ME), neutral detergent fiber (NDF) content and forage nutritional value (FNV) of four dominant species in June, July, August and September (means ± standard error). Means with different letters in the same month differ from each other (*P<* 0.05).

In general, with the progress of summer, FNV, CP, IVTD and ME of all four plant species plant decreased, while NDF increased. The rate of increase of each variable differed among species, mainly because the phenological development of each species differed (**Figure 4**; **Table S3**).

The greatest change of CP content occurred between June and September for all four plant species, with the largest change in *P. annua*, from 15.8 to 9.0% at 7.5 sheep/ha, and the smallest change in *P. fruticosa*, from 9.4 to 5.9% at 0 sheep/ha (**Figure 4**). The greatest change of IVTD occurred between June and September for all four species, with the largest change in *P. annua*, from 70.1 to 51.4% at 7.5 sheep/ha, and the smallest change in *P. fruticosa*, from 64.3% to 55.3% at 0 sheep/ha (**Figure 4**). The greatest change of ME occurred between June and September for all four species, with the largest change in *P. annua*, from 9.9 to 6.7 MJ/kg at 7.5 sheep/ha, and the smallest change in *A. adsurgens*, from 8.4 to 6.9 MJ/kg at 0 sheep/ha (**Figure 4**). The greatest change of NDF content occurred between June and September for all four species, with the largest change in *P. annua*, from 46.5% to 66.7% at 7.5 sheep/ha, and the smallest change in *P. fruticosa*, from 66.4% to 71.1% at 0 sheep/ha (**Figure 4**).

### Effects of stocking rate and precipitation on forage nutritional yield

**Figure 5:**
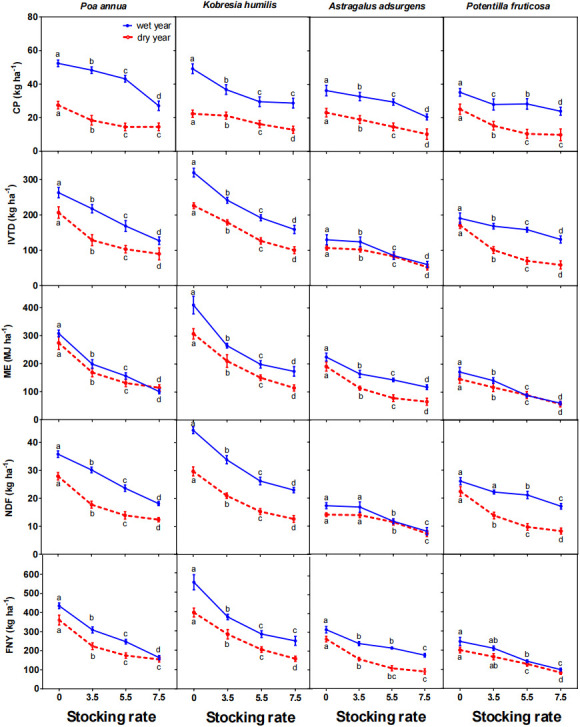
Effects of stocking rate on forage nutritive yield (FNY), crude protein (CP) yield, *in vitro* true digestibility (IVTD), metabolizable energy (ME), neutral detergent fiber (NDF) and forage nutritional value (FNV) yield of four dominant species in a wet year (2019) and a dry year (2020) (means ± standard error). Means with different letter in the same year differ from each other (P< 0.05).

Grazing (G) and precipitation (Y) and the interaction between them affected the FNY in all four plant species (*P*<0.05) (**Table S4**). The FNY of the four species were greater (*P*<0.05) in the wet year than in the dry year, and decreased with increasing grazing intensity (**Figure 5**; **Table S4**).

**Figure 6:**
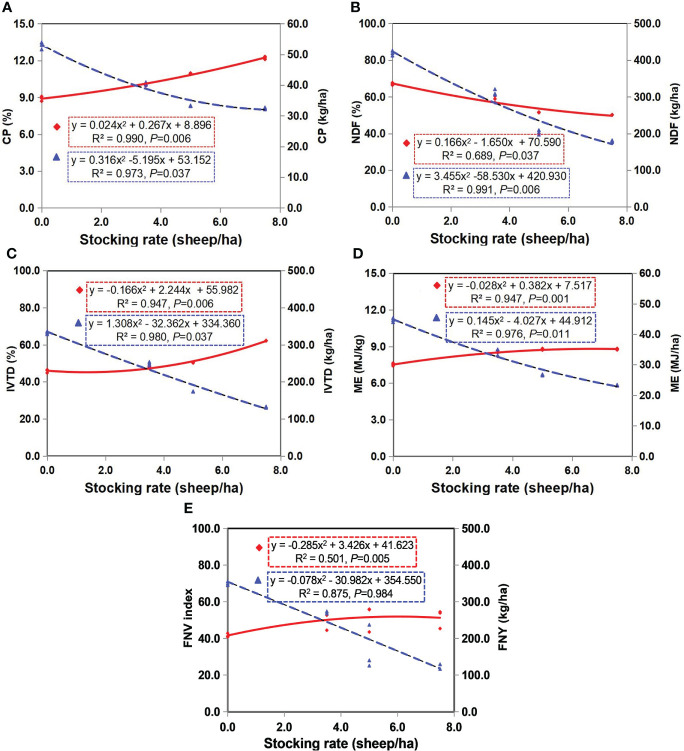
Relationship between stocking rate and forage nutritive value (FNV; left y-axis, %) and forage nutritive yield (FNY; right y-axis, kg ha^-1^) for four dominant species in two years (2019 and 2020): **(A)** crude protein (CP), **(B)** *in vitro* true dry matter digestibility (IVTD), **(C)** neutral detergent fiber (NDF), **(D)** metabolizable energy (ME) and **(E)** forage nutritional value (FNV). Solid lines denote concentrations (♦), and long-dashed regression lines denote yields of herbage nutritional parameters (▲) (yields = concentrations × dry matter of above-ground biomass).

The FNV improved as the concentrations of CP, IVTD, and ME increased and of NDF decreased with an increase in grazing intensity (**Figure 6**). These effects are illustrated in **Figure 6**, which highlights the linear relationship between grazing intensity and NDF (**Figure 6B**), and the quadratic relationships between grazing intensity and CP (**Figure 6A**), IVTD (**Figure 6C**), and ME (**Figure 6D**). The CP content (%; **Figure 6A**), IVTD (%; **Figure 6C**), and ME yield (MJ/kg; **Figure 6D**) were related inversely to CP content (kg/ha), IVTD (kg/ha) and ME content (MJ/ha). IVTD (%) and ME yield (MJ/kg) increased significantly with a decline in NDF content (%; *P_IVTD_* = 0.006; *P_ME_* = 0.001).

## Discussion

### Inter-annual patterns of grazing effects on forage nutritional value

Consistent with previous studies (Mertens, 1994; Schulze et al., 1994; Van Soest, 1994; Huston, 1997; Snyman, 2002; Garcia et al., 2003; Li et al., 2017), results in the present study indicated that grazing increased FNV of the four dominant species. This is because herbivores feed on the above-ground mature stems and leaves of plants, which stimulate the regeneration and growth of forage, resulting in new tissue. The maturation and lignification process of forage are delayed, and most of the nitrogen stored in plant stems and roots is transferred to the new tissues of plants, that is, twigs and leaves (Mertens, 1994; Schulze et al., 1994; Huston, 1997; Fanselow et al., 2011), Furthermore, grazed pasture has a higher nitrogen absorption efficiency than non-grazed pasture, which enhances FNV (Garcia et al., 2003; Schiborra et al., 2009; Bai et al., 2012; Li et al., 2017). The effect of herbivore grazing on alpine grassland resulted in an increase in nitrogen content with a concomitant decrease in ANPP (Long et al., 2003). Nitrogen from dung and urine from grazing livestock were reported to have a positive effect on nitrogen content in shrubs of desert grassland and steppe grassland grass species, and accelerate the mineralization rate of senescent plant litter in surface soil. Subsequently, soil mineral nitrogen increased the nitrogen content in grasses and some shrubs (Jiao et al., 2011; McCarthy et al., 2013).

The present study also demonstrated differences in the FNV among plant species in response to grazing. Grazing clearly increased the FNV of all four species, as CP content, IVTD and ME yield increased, but had a lesser effect on *P. fruticosa* than the other plant species. Based on the ranking of the slopes for the regression lines of ME yield on stocking rate, Tibetan sheep preferred *P. annua* in a wet year and *A. adsurgens* in a dry year. *K. humilis* is the dominant species on the Tibetan Plateau due to its high biomass production, early regrowth, wide distribution and strong resistance to both over-grazing and extreme climatic conditions. It is very palatable and of particular importance in alpine grasslands in the spring, a critical period for livestock survival and also when most lambings occur. This was demonstrated by Long et al. (2003) who reported a 30% decrease in dry matter intake and body weight in sheep during spring. *P. annua* is highly palatable and readily consumed by grazing sheep, resulting in new biomass being produced with a high FNV. This plant should be consumed during autumn and early winter to avoid the loss of leaves and reduction in nutritive value. However, the increased nutritional value of this palatable plant leads to increased consumption by sheep. *P. fruticosa* is a shrub, and grazing often reduces the apical dominance, causing more branching. The new leaves that are produced are smaller, thinner and have higher nitrogen and lower fiber content due to increased intra-plant competition (Miao et al., 2015). However, *P. fruticosa* has a relatively low palatability, leading to low consumption by sheep. This could explain why grazing had little effect on the nutritive value, as only CP content increased, with no effect on IVTD and ME yield. The CP content and ME yield of *P. fruticosa* are low, so its contribution to the nutrient and energy intakes of grazing livestock is limited.

The legume *A. adsurgens* is capable of nitrogen fixation with symbiotic bacteria. The N content of this legume is higher and the mechanisms by which they acquire N differ from the three other species. Due to the high N and low fiber contents, this plant species is highly nutritious and consumed, resulting in new biomass, with a high FNV being produced. The increased nutritional value of this palatable forage leads to an increased consumption by sheep. *A. adsurgens* is often mixed with *P. annua*, *K. humilis* and *P. fruticosa* in high altitude alpine grassland and is generally grazed only in summer. During this time of the year, sheep traditionally migrate from the low alpine grassland to a higher elevation. The present study indicated that *A. adsurgens* contains high crude protein concentrations and provides substantial energy. In mixed plant communities, this species can be very useful in providing extra dietary nitrogen so that other forages can be utilized more efficiently as energy sources. The present study demonstrated that different plant species differ in their adaptive strategies against grazing, including grazing-tolerance (i.e., ability of rapid regrowth, as *P. annua* and *K. humilis*), grazing avoidance (i.e., low palatability, as *P. fruticosa*) or leguminous nitrogen fixation (as *A. adsurgens*).

In the present study, the FNV in the wet year was greater than in the dry year, which is consistent with previous studies in grass species and shrubs (Schulze et al., 1994; McCarthy et al., 2013; Mekuria & Aynekulu, 2013). In alpine grasslands, species growth is limited by water availability (McCarthy et al., 2013). In wet years, high CP content in herbage can be attributed to the efficient use of nitrogen by plants due to accelerated mineralization (Austin et al., 2004), and the high capacity of species to assimilate nitrogen (Wen et al., 2018). Water stress causes early maturity, an increase in fiber content, and a reduction in digestibility of herbage and in FNV. Consistent with previous studies (Schiborra et al., 2009; McCarthy et al., 2013), we also found that the impacts of grazing on FNV were dependent on precipitation. During the wet year, grazing increased the FNV of *P. annua* and *K. humilis*, but there was no effect during the dry year, suggesting that soil water content was more important in determining the nutritional value of plant species than grazing. Because nitrogen absorption is limited by the soil water content (Fanselow et al., 2011; Giese et al., 2013), the nitrogen content in new biomass was lesser in the dry year than in the wet year. It is possible that *P. fruticosa* has high water use efficiency, and, consequently precipitation did not affect the nutritional values.

### Seasonal patterns of forage nutritional value due to grazing

The FNV of the four species decreased with time, while forage maturation accelerated after June, which was also reported in previous studies (Bailey, 2004; Mekuria & Aynekulu, 2013). As species stop growing, maturation and lignification set in, with an increase in cellulose, hemicellulose and lignin and a decrease in cellular substances, such as proteins (Müller et al., 2014). These patterns are associated with a decrease in ME, IVTD and CP and an increase in NDF. It is important to note that the decline in nutritive value in grasses occurs rapidly after its growth, therefore, intensive grazing in the early summer months in alpine meadows may be advisable. In addition, grazing of N rich forages, particularly in the summer months, should help meet the requirement for rumen degradable protein and improve animal performance. Long et al. (2003) reported that blood urea concentration in sheep was greatest during August and then declined to a minimum value in February (Long et al., 2003). This reflects the poor protein balance of sheep after October.

The relative decrease in ME yield with plant maturity was slower than that of CP, which implies that N may be a more limiting factor in sheep nutrition than energy. A previous study reported that a diet containing CP at 13.2% DM could be fully utilized by 35 kg grazing sheep (Vesk et al., 2004). In the present study, the average CP content of the four dominant species at the 4 stocking rates ranged between 13.5 and 14.9% DM in the wet year and between 10.2 and 10.8% DM in the dry year. Based on these values, CP concentration of the forage was below the optimal level for grazing sheep during the dry year; therefore, supplementing grazing sheep with dietary protein at this time is recommended.

### Effects of stocking rate and precipitation on forage nutritional yield

FNY, determined by ANPP and FNV, is a key parameter for livestock production in alpine grassland ecosystems. Consequently, the FNY in response to grazing or precipitation was predictably similar to the ANPP response. As grazing intensity increased, the increase in FNV did not compensate for the decrease in ANPP, resulting in a substantial decrease in FNY, which was consistent with the results of a previous study (Buxton, 1996). This provides important implications for future pasture management in balancing ANPP and FNV. This study accounted for the contributions of ANPP and FNV to FNY, and, as a result, the conclusions are more comprehensive and reliable than those made in earlier studies (Schiborra et al., 2009). As ANPP decreases with increasing grazing intensity, long-term grazing reduces the sustainability of alpine grassland in the QTP. Future climate change is likely to increase extreme drought and rainfall events (Easterling et al., 2000), which will affect ANPP and FNV. Increased drought severity and over-grazing may accelerate the conversion of forage from high to low quality and from high to low yield (Yang et al., 2018). Our research provides a meaningful guidance for improving the management of alpine grassland ecosystems on the QTP to prevent these negative changes.

Ren et al. (2008) reported that ANPP, FNV and FNY were determined mainly by the dominant plant species, rather than by species diversity, and Hodgson (1982) added that the nutritional levels of a plant community are determined by the most productive species because they are well adapted to the environment. According to Pakeman (2004) and Yang et al. (2018), dominant species play an important role in maintaining community stability, while species composition is dependent on the many different functional traits from all species, from dominant to opportunistic. In the present study, the four species comprised a very high percentage (88.6%) of the pasture, and, consequently, affected the ANPP, FNV and FNY more than other plant species. In future studies dominant species can be used as effective indicators for predicting the FNV and FNY of pasture. Vegetation dynamics and grassland stability can be predicted by different herbivore intensities and monitoring over successive years.

## Conclusions

The present study demonstrated that the interaction between livestock density and precipitation was a major driver of ANPP. Grazing regimes explained at least 76% of the variation of ANPP, while the effect of precipitation explained between 5% and 12%. Grazing increased FNV, but decreased ANPP and FNY. In non-grazed plots, the yield of CP/ha declined sharply (18%-55%) in response to drought, but ME yield was not affected. This study highlights that the effects of livestock density on alpine meadow pasture is contingent on rainfall, and that studies designed to understand responses of pasture should incorporate co-occurring drivers of change, such as herbivore density regimes and precipitation.

## Data availability statement

The original contributions presented in the study are included in the article/supplementary material. Further inquiries can be directed to the corresponding authors.

## Author contributions

XY: Data curation, formal analysis, methodology, writing-original draft (supporting). CL: Methodology, conceptualization, writing review. AA, AT: Writing-review & editing, formal analysis. AD: Methodology (supporting), writing-review & editing. YB: Conceptualization, methodology, funding acquisition, supervision, writing-review & editing. All authors contributed to the article and approved the submitted version.

## Funding

This work was supported by grants from The Open Project of State Key Laboratory of Plateau Ecology and Agriculture, Qinghai University (2022-ZZ-04), Wetland Ecological Benefit Compensation Project of Taoheyuan National Wetland Park (2021-007), National Key R&D Program of China (2021YFC3201600) and Sichuan Agriculture University Scientific Research Start-up Fund (031-2122996042).

## Acknowledgments

We would like to thank our colleagues for contributing to data collection, the conceptualization and for commenting on the manuscript.

## Conflict of interest

The authors declare that the research was conducted in the absence of any commercial or financial relationships that could be construed as a potential conflict of interest.

## Publisher’s note

All claims expressed in this article are solely those of the authors and do not necessarily represent those of their affiliated organizations, or those of the publisher, the editors and the reviewers. Any product that may be evaluated in this article, or claim that may be made by its manufacturer, is not guaranteed or endorsed by the publisher.
